# A Case of Pregnancy in a Female, in Whom the Menstrual Function Had Been for Some Years Suspended

**Published:** 1857-09

**Authors:** O. C. Gibbs

**Affiliations:** Frewsburg, New York


ART. V.—*A Case of Pregnancy in a Female, in whom the Menstrual Function had been for some years suspended.* By O. C. GIBBS, M.D., of Frewsburg, New York.

IT is a well-established physiological fact, that menstruation only occurs when there exists a certain amount of ovarian integrity, and that pregnancy can only exist in women capable of menstruation. In some rare instances girls have become pregnant before the establishment of the menstrual function, and nursing women not unfrequently become so before the return of the catamenia, suspended by a previous pregnancy. In such cases it is supposed that the requisite ovarian integrity exists to determine menstruation; that the first mature ovum, which would determine the commencement of the menstrual function in the one case, and its return in the other, becomes impregnated previous to its escape, which escape would have developed the catamenia.

A case occurred to me, not long since, which, though it may not militate against the well-established physiological doctrines hinted at above, was yet well calculated to deceive in regard to its true nature, and hence is worthy of record. It is not always easy to determine the existence of pregnancy, and sometimes the determination is not made because the true condition is not suspected. It is not a little mortifying to the physician, in such cases, to express a false diagnosis, and having just passed the ordeal of such a mortification, I am disposed to make a record of the sources of my mistake, that others may be more circumspect.

February 2d. I was requested to visit Mrs. A., who resided some twelve miles off. I complied with the request, and found the patient, who was a stranger to me, able to be about the house most of the time, and perform some light work. She complained of extreme fulness and pressure about the bowels and chest; respiration was short and hurried; slight exertion produced dyspnœa; a full meal increased the sensation of oppression, which was occasionally relieved by vomiting, the latter occurring without nausea. She had frequent desires to urinate, being compelled to rise from six to a dozen times a night for that purpose. The horizontal position was insupportable, and she could sleep only by being bolstered up to nearly a sitting posture. Though pale, she was quite corpulent; yet it was evident her abdomen was greatly distended by some foreign substance.

The history of the case was this: She was thirty-two years of age; had been married ten years; had never borne children; never had an abortion; never had unnatural uterine hemorrhage. From the commencement of her menstrual period until about three months subsequent to her marriage, she had been quite regular in her catamenia. From this time she became quite irregular, both as to the time and quantity of her menstrual flow, the time varying from six weeks to six months, and the quantity unusually small. For the first seven years succeeding her marriage, the catamenial irregularity was as stated above. During the eighth year she menstruated but once, and during the ninth and tenth not at all. Hence, it will be seen that, in this case, ovarian integrity, which determines menstruation, had been gradually waning for nearly ten years. During the last three years my patient had menstruated but once very scantily, and that once about two years since. The abdomen had been slowly but steadily enlarging for five years, though more rapidly during the last few months. She had been, for the last two years, at different times, under the treatment of different physicians, without apparent benefit, for the same symptoms, for the relief of which she now consulted me. So much for the history and general symptoms of the case.

In view of the irregularity of the menses and their total suppression for about two years, pregnancy was supposed impossible, and hence, not considered in the further examination of the case. It seemed to me evident that the case was one of ascites, encysted dropsy of the ovaries, or an accumulation of menstrual or other fluid within the uterus, in consequence of impermeability of the cervix uteri. With the patient lying upon the back, percussion over the abdomen yielded a dull sound in front, with resonance near the spine; fluctuation was distinct, and after a careful examination I could detect no solid tumor in the abdominal cavity. Dulness on percussion, elasticity, and fluctuation, characterized the abdominal enlargement. The case was evidently not one of ascites.

A digital examination of the uterus per vaginam, was made, but in consequence of the elevated position of that organ and the corpulent condition of the patient, no positive results were obtained. I could not reach the os uteri, but it was evident that the womb was much distended. This fact alone inclined me to the opinion that I had to deal with a case of impermeability of the cervix uteri, and a consequent retention and accumulation of menstrual or other fluid within the uterine cavity. I was not wholly satisfied with my diagnosis, and acknowledged to the patient my indecision; but being earnestly pressed by the friends for a definite opinion, I stated that there was an unnatural secretion of fluid, a dropsical accumulation, which in my opinion was partly within and partly external to the uterus. As the sequel will show, this statement was true so far as it went, but it was not the whole truth.

My remedial efforts were directed wholly to the mitigation of the more prominent and distressing symptoms, and were not wholly without utility. I saw the patient at intervals of a week or ten days, and at my third visit again made a thorough examination of the case, with the hope of more satisfactory results. In this I was disappointed, and consequently pursued my former plan of treatment, waiting patiently for further developments in the case.

About six weeks after my first visit, I made a third examination per vaginam, and accidentally detected ballottement. This diagnostic symptom was certainly unexpected, but it was distinct and unmistakable. It probably might have been detected at first, had it been sought for; but, in view of circumstances previously stated, pregnancy was supposed impossible, and consequently no means were taken to detect it. I again examined the abdomen externally, but no manipulation which I could bring to bear upon the case gave evidence of a solid body within; yet to my great mortification, and the patient's great surprise, I unhesitatingly pronounced her pregnant.

About a month after this, and two and a half from the time I first saw the patient, she was taken in labor and was delivered of a male child, to all appearances fully developed as to time, yet weighing only about three and a half pounds. There was nothing peculiar about the labor except that the liquor amnii was excessive, there being probably from six to eight quarts. There was considerable fluid remaining in the abdomen after labor, giving evidence that the case was complicated with ascites. The woman recovered, with no unpleasant symptoms, the ascites passing away without treatment. The child died at two months old, of capillary bronchitis; and up to the present time the menstrual function has not returned, though the woman seems in perfect health.

This case presents some points of peculiarity, which it seems to me were well calculated to deceive in the outset, in regard to its true nature. The patient was a lady of refinement, and of more than ordinary intelligence, and I think the utmost reliance can be placed upon her statements. I certainly can appreciate no motive which she could have for deception, and her statements were confirmed by her husband, also by her sisters.

It is not impossible but that the ova matured and were expelled regularly, in this case, without producing the ordinary catamenial products; but I rather incline to the opinion that such maturation and expulsion occurred at irregular and uncertain intervals. Be this as it may, I am indisposed to add additional speculations. Having made a record of the case as it presented itself, I submit it without further comment.

